# Long-term prognostic value of Murray law-based quantitative flow ratio in jailed left circumflex coronary artery after left main crossover stenting

**DOI:** 10.1038/s41598-023-30991-4

**Published:** 2023-03-16

**Authors:** Lieyou Li, Zhihai Feng, Lin Zhang, Huang Chen, Hong Zheng, Huizhong Lin, Qiong Jiang, Yunlin Lin, Lianglong Chen, Lin Fan

**Affiliations:** Department of Cardiology, Institute of Coronary Heart Disease, Fujian Medical University Union Hospital, Xinquan, Road 29#, Fuzhou, 350001 Fujian China

**Keywords:** Cardiology, Interventional cardiology, Computational biology and bioinformatics, Computational models

## Abstract

We aimed to evaluate the impact of new Murray law-based QFR of jailed left circumflex coronary artery (LCx) on long-term clinical outcomes after left main coronary artery (LM) simple crossover stenting. 164 patients who underwent LM-to-left anterior descending coronary artery simple crossover stenting and had appropriate angiographic view of LCx for QFR computation were enrolled. The primary clinical outcome was the 5-year target lesion failure (TLF), defined as a composite of cardiac death, a target vessel myocardial infarction or target lesion repeat revascularization. The mean QFR of the LCx after LM stent implantation was 0.88 ± 0.09, and 29 patients (17.7%) had a low QFR (< 0.80), which was significantly associated with a higher 5-year rate of TLF when compared with the high QFR group (27.6% vs. 6.7%; HR: 4.235; 95% CI 1.21–14.95; p = 0.0015). The 5-year LCx ostium-related TLR rate in the low QFR group was also higher (17.2% vs. 3.0% in the high QFR group; HR: 6.07, 95% CI 1.63–22.59, p = 0.002). In a multivariate Cox regression analysis, a low QFR in the LCx after LM stenting was an independent predictor of the 5-year TLF rate (HR: 3.21, 95% CI 1.21–8.53; p = 0.019). ROC analysis showed that QFR a negative predictive value (NPV) of 89.6% ([AUC] 0.73, 95% CI 0.58–0.88, p < 0.05), the cutoff point is 0.85. The patients with a low QFR (< 0.80) in jailed LCX after LM simple crossover stenting had worse 5-year outcomes than those with a high QFR. Conversely, a QFR ≥ 0.85 of jailed LCx could serve as a good predictor of low risk of adverse outcome in LCx ostium. The QFR computation of the jailed LCx may be helpful to determine whether an additional procedure is required for the jailed side branch.

## Introduction

Left main (LM) distal bifurcations represent one of the most challenging lesion subsets in the field of percutaneous coronary intervention (PCI). Currently, a provisional approach is still preferred for the majority of LM bifurcations^1–5^. Nevertheless, high risk of angiographically jailed SB persists after main vessel stent implanted. In clinical practice, additional kissing balloon inflation or kissing balloon inflation after bailout stenting is generally used to treat the jailed side branches (SB). However, routine usage of such technique cannot result in more benefits for the patients with LM bifurcation lesion but potentially increase the additional risk of adverse clinical events. Meanwhile, such anatomy-based angiographic stenosis cannot accurately reflect functional significance^6^.

Fractional flow reserve (FFR), as a 'gold standard' of coronary stenosis functional severity indices, has been proved to be safe and feasible for assessing the jailed SB from LM or non-LM bifurcation. Previous studies also showed that FFR-guided SB intervention strategy is beneficial to improve clinical prognosis, and those patients with lower FFR (< 0.8) in a jailed left circumflex coronary artery (LCx) after LM crossover stenting had poorer long-term clinical outcomes^7–10^. Thus, FFR can be used as a reliable tool for operators to make a reasonable decision for the treatment of jailed SB. However, its application in clinical real world is still limited to requirements for intracoronary pressure wire and adenosine inducing hyperemia. Meanwhile, SB access with a pressure wire after main vessel stenting is sometimes difficult and has a potential risk of SB dissection^11,12^. In the last few years, several methods of angiography-based FFR have presented to overcome invasive FFR limitations. Especially, of which quantitative flow ratio (QFR) has been proved to have good diagnostic accuracy for functional ischemia compared with FFR in previous studies^13–18^. The Murray law-based QFR is a novel approach to assess the functional significance of coronary artery stenosis for the main vessel and all major side branches, simultaneously^18^. However, its effectiveness in assessment of functionally side branch compromise and impact on long-term prognosis has not been validated in bifurcation lesions after main vessel stent implanted. Here, we evaluated the long-term prognostic value of QFR of a jailed LCx after LM crossover stenting.

## Methods

### Patients selection

The patients who underwent LM-to-left anterior descending coronary artery (LAD) simple crossover stenting without any additional procedures were retrospectively screened from the Coronary Angiography and Angioplasty Registry Database of Fujian Medical University Union Hospital, registered from January 2013 to June 2016. The inclusion criterions were as follows: (1) angiographically visible de novo coronary artery disease in the distal LM or ostial LAD, and without any significant ostial LCx disease; (2) the reference vessel diameter of the LCx should be ≥ 2.5 mm; (3) suitable angiographic view with minimal vessel overlap for QFR computation. The patients were excluded according to following conditions: pre-treatment history of an ostial LCx; significant non-ostial LCx lesion; infarct-related artery or visible thrombus in target vessel; left ventricular ejection fraction < 35%; a major life-threatening illness such as end-stage of malignent tumor; primary myocardial disease as hypertrophic cardiomyopathy, dilated cardiomyopathy. The study protocol was approved by the Ethics Committee of Fujian Medical University Union Hospital (2021KY147) and conformed to the principles outlined in Declaration of Helsinki. All patients gave written informed consent.

### Analysis of QFR and quantitative coronary angiography

QFR was analyzed by a certified analyst who blinded to clinical outcomes according to standard operating procedures by using the AngioPlus software (Pulse Medical Imaging Technology, Shanghai, China). The analyst chose the angiographic view with minimal vessel overlap on both the interrogated vessel and its SBs ostium as the optimal angiographic view. The QFR was calculated as described by a previous study^18^, which showed detailly in the appendix.

### Percutaneous coronary intervention procedures

Left main coronary artery simple crossover stenting procedures were performed according to standard techniques: (a) wiring to main branch or both branches if necessary; (b) predilatation of the main branch prior to stenting; (c) main branch stenting using a stent diameter according to the distal main branch reference; (d) proximal optimisation technique (POT). After POT, the procedure can be stopped. Whether an additional procedure (side branch dilation, kissing balloon inflation or re-POT) was needed for the side branch at the operators’ discretion. All patients were treated with a loading dose of aspirin (300 mg) and P2Y12 receptor inhibitor (clopidogrel 300 mg, or Ticagrelor 180 mg) before the percutaneous coronary intervention (PCI). After the procedure, aspirin was prescribed for lifetime (100 mg daily), with P2Y12 receptor inhibitors for at least 12 months (clopidogrel 75 mg daily, or Ticagrelor 90 mg twice daily).

### Follow up and endpoints definition

Clinical follow-up was conducted by independent trained reviewers, clinical endpoint events happened within the first 5-year after PCI was recorded from the review of hospital charts, or discharge summary review, clinical visits or telephone interviews. The primary endpoint was target lesion failure (TLF), defined as the composite of cardiac death, target vessel myocardial infarction (MI), or target lesion repeat revascularization (TLR). The secondary endpoints including: (1) TLR of LCX ostium (LCXos), (2) all-cause death (noncardiac or cardiac cause), (4) MI (target vessel or nontarget vessel) (3) target-vessel failure (TVF)—composite endpoint of death from a cardiac cause, target vessel MI, target vessel revascularization (TVR), (5) Repeat revascularization (TVR or TLR), and (6) major adverse cardiac event-composite endpoint (MACE) was composed of all-cause death, MI, any repeat revascularization.

Death was defined as cardiac cause unless definite non-cardiovascular causes could be identified. According to the fourth universal definition of MI, diagnosis of MI required a combination of symptoms, electrocardiographic changes, and significant increase in cardiac troponin values (≥ 99th percentile upper reference limit)^19^. TLR was defined as any repeat revascularization procedure (PCI or coronary artery bypass surgery) for the original target lesion site, involving the stent and within 5 mm of proximal and distal margins of the stent and LCx ostium^20^.

### Intra- and inter-observer analysis

Intra-observer and inter-observer agreement in QFR computation was performed in 50 randomly selected patients, angiographic view of selected vessels was reanalyzed by the same analyst 7 days later and by a second qualified analyst, following the same standard operation procedure and being blinded to each other or to the previous computational results.

### Statistical analysis

Dichotomous and categorical variables were presented as frequencies and percentages, and differences were assessed using chi-square tests and Fisher’s exact tests. Continuous variables were described as mean and standard difference, and differences among groups were assessed using the Mann–Whitney U test. All covariates that were either statistically significant (p < 0.20) on univariate analysis or clinically relevant were taken into a multivariate Cox regression model. Adjusted hazard ratios (HRs) were estimated by Cox regression model and were presented with 95% confidence interval (CI). Cox proportional hazard models were also applied to compare the clinical events according to the LCx QFR after stenting. Survival curves were constructed using Kaplan–Meier estimates for the time to the clinical endpoint and between-group differences was compared by the log-rank test. For the purpose of the sensitivity analysis, a propensity score was estimated by fitting a logistic-regression model that adjusted for age, multivessel disease, distal reference vessel diameter of LM-LAD, distal reference vessel diameter of LCx. 1:3 pair matching between the two groups was performed by nearest neighbor matching without replacement. The same analysis was performed for the cohorts after propensity score matching (PSM). All reported p values were 2-sided and p < 0.05 was considered to be statistically significant. All analyses were performed with the SPSS statistical software (version 23.0, IBM, Chicago, USA) and R package.

### Ethics approval

The present study was approved by the Clinical Research Ethics Committee of Union Hospital, Fujian Medical University, Fuzhou, Fujian province, China.

## Results

### Reproducibility in repeated QFR analysis

Repeated QFR computation was performed in 50 vessels. Intra-observer and inter-observer variability in QFR was 0.000 ± 0.012 and 0.002 ± 0.011, respectively.

### Characteristics of the patients and lesions

**Figure 1:**
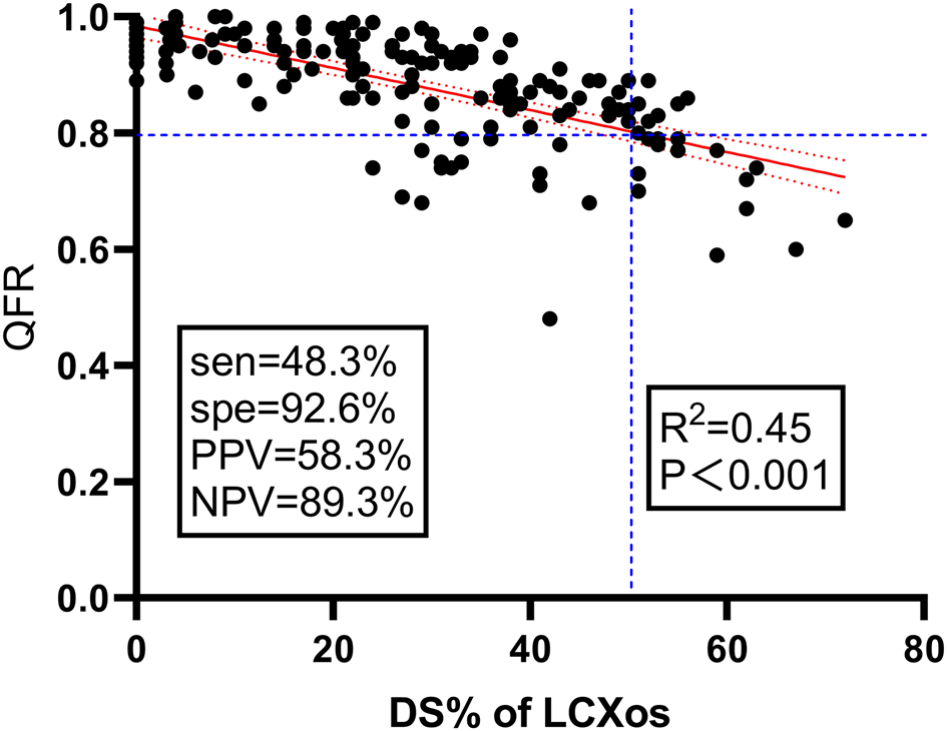
Correlation between QFR and DS% of Jailed LCx after LM simple crossover stenting. *QFR* quantitative flow reserve, *DS%* percentage of diameter stenosis, *Sen* sensitivity, *Spe* specificity, *NPV* negative predictive value, *PPV* positive predictive value, *LCXos* the ostium of left circumflex coronary artery, *LM* left main coronary artery.

164 patients who underwent LM to LAD simple crossover stenting were eligible for enrolment and completed QFR computation.The baseline demographics and clinical characteristics of 164 patients according to the QFR are shown in Table 1. Baseline characteristics were comparable except for older age (67.41 ± 9.73 vs. 61.97 ± 10.5, p = 0.008) in the high QFR group. Table 2 showed the procedural results of the study population according to the QFR, the reference vessel diameter, minimal lumen diameter, percentage of diameter stenosis (DS%) and lesion length of the LM-to-LAD before PCI were comparable between the two groups (all p˃0.05). Compared with the high QFR patients, the reference vessel diameter, minimal lumen diameter of LCx was smaller (2.77 mm (2.50, 2.90) vs. 2.92 mm (2.60, 3.20), p = 0.039; 2.20 mm (1.8, 2.55) vs. 2.57 mm (2.20, 3.00), p = 0.001, respectively) in the low QFR patients, DS% of LCX was accordingly higher (21.97% (5.0, 42.0) vs. 13.47% (0, 23.0), p < 0.001). There was no difference between the two groups in the stent length and diameter. After LM stent implantation, no difference was found as to the reference vessel diameter, minimal lumen diameter, DS% and QFR of LM-LAD between groups (all p > 0.05). In comparison with the high QFR patients, the mimimum lumen diameter of ostial LCx in the low QFR group was smaller (1.54 mm (1.10, 1.80) vs. 2.22 mm (1.80, 2.70), p = 0.001), the DS% of ostial LCx was accordingly higher (45.93% (13.60, 57.0) vs. 24.32% (11.00, 37.00), p = 0.001), and the QFR of the jailed LCx was significantly lower (0.72 (0.68, 0.76) vs. 0.92 (0.87, 0.96), p = 0.001). After PSM, no statistical difference was observed between the two groups with the exception of minimal lumen diameter and DS% in the ostium of LCx (Table S1). Besides that, correlation analysis pointed out that a good correlation between QFR and post-stent DS% of the jailed LCXos was found (R^2^ = 0.45, p < 0.001) (Fig. 1).

**Table 1:** Baseline characteristics of patients.

	All patients (n = 164)	High QFR (n = 135)	Low QFR (n = 29)	P value
Age (years)	66.45 ± 10.06	67.41 ± 9.73	61.97 ± 10.5	0.008
Men, n (%)	144 (87.8)	119 (88.1)	25 (86.2)	0.758
Hypertension, n (%)	103 (62.8)	86 (63.7)	17 (58.6)	0.674
Diabetes mellitus, n (%)	68 (41.5)	50 (37)	14 (48.3)	0.297
Insulin treatment, n (%)	20 (12.2)	18 (13.3)	2 (6.9)	0.533
Hyperlipidemia, n (%)	51 (31.1)	41 (30.4)	10 (34.5)	0.664
Current smoking, n (%)	101 (61.6)	82 (60.7)	19 (65.5)	0.679
Previous MI, n (%)	59 (36)	46 (34.1)	13 (44.8)	0.292
Overweight (BMI ≥ 24), n (%)	80 (48.8)	67 (49.6)	13 (44.8)	0.686
Ejection fraction (%)	65.05 (55.42, 68.88)	65.8 (57.3, 68.9)	61.1 (48.0, 67.9)	0.214
NT-proBNP (pg/ml)	147 (56.0, 527.5)	145.0 (56.0, 548.0)	156.0 (62, 449.5)	0.853
Clinical presentation				
Stable angina, n (%)	29 (17.7)	25 (18.5)	4 (13.8)	0.789
Acute cornary syndrome, n (%)	135 (92.3)	110 (81.5)	25 (86.2)	
STEMI	22 (13.4)	18 (13.3)	4 (13.8)	
Non-STEMI	56 (34.1)	42 (31.1)	14 (48.2)	
Multivessel disease, n (%)	80 (48.8)	61 (45.2)	19 (65.5)	0.065

**Table 2:** Lesion and procedural details.

Baseline	All patients (n = 164)	High QFR (n = 135)	Low QFR (n = 29)	P value
LM-LAD				
Reference vessel diameter, proximal (mm)	3.25 (3.0, 3.5)	3.26 (2.90, 3.50)	3.18 (3.0, 3.5)	0.370
Reference vessel diameter, distal (mm)	2.57 (2.30, 2.80)	2.54 (2.30, 2.80)	2.71 (2.35, 3.0)	0.105
Minimal lumen diameter (mm)	1.37 (1.0, 1.60)	1.37 (1.00, 1.60)	1.38 (1.05, 1.65)	0.894
Diameter stenosis (%)	50.80 (42.25, 62.0)	51.71 (43.0, 63.0)	46.52 (38.0, 59.5)	0.127
LM-LAD lesion length (mm)	23.20 (12.5, 27.72)	22.65 (12.10, 27.50)	25.79 (14.3, 34.1)	0.367
LCx				
Reference vessel diameter, distal (mm)	2.89 (2.60, 3.10)	2.92 (2.60, 3.20)	2.77 (2.50, 2.90)	0.039
Minimal lumen diameter (mm)	2.51 (2.15, 2.88)	2.57 (2.20, 3.00)	2.20 (1.8, 2.55)	0.001
Diameter stenosis (%)	14.98 (2.03, 23.75)	13.47 (0, 23.0)	21.97 (5.0, 42.0)	0.004
LM-LAD stent				
Stent length (mm)	25.10 (18.0, 30.0)	25.11 (18.0, 30.0)	25.02 (18.0, 31.5)	0.962
Stent diameter (mm)	3.51 (3.5, 4.0)	3.51 (3.5, 4.0)	3.50 (3.0, 4.0)	0.853
After LM-LAD stenting				
LM-LAD				
Minimal lumen diameter (mm)	2.66 (2.22, 3.10)	2.64 (2.20, 3.10)	2.79 (2.40, 3.20)	0.277
Diameter stenosis (%)	13.53 (2.0, 21.75)	14.09 (2.90, 22.0)	10.95 (0, 16.5)	0.213
LM-LAD QFR	0.94 (0.92, 0.98)	0.94 (0.92, 0.98)	0.94 (0.92, 0.98)	0.625
Ostial LCx				
Minimal lumen diameter (mm)	2.10 (1.60, 2.59)	2.22 (1.80, 2.70)	1.54 (1.10, 1.80)	0.001
Diameter stenosis (%)	28.14 (15.0, 41.0)	24.32 (11.00, 37.00)	45.93 (13.60, 57.0)	0.001
LM-LCx QFR	0.88 (0.84, 0.95)	0.92 (0.87, 0.96)	0.72 (0.68, 0.76)	0.001

### Clinical impact of the QFR in jailed LCx after LM crossover stenting

The median follow-up time of all enrolled patients was 5.4 years. As showed in Table 3, a low QFR of LCx after LM stenting was an independent predictor of the 5-year TLF rate (HR 3.21, 95% CI 1.21–8.53; p = 0.019). However, the pre-stent and post-stent vessel parameters including DS% and minimal lumen diameter were not associated with 5-year TLF. Especially, minimal lumen diameter and DS% of ostial LCx were also not related with 5-year TLF rate in multivariate cox proportional hazard analyse. In PSM population, a low QFR of LCx remained a risk factor for 5-year TLF and TLR of osLCX (all p < 0.05) (Tables S3 and S4).

**Table 3:** Univariate and multivariate cox proportional hazard analyses for 5-year TLF.

	Univariate		Multivariate	
	HR (95% CI)	P value	HR (95% CI)	P value
Age	1.012 (0.97, 1.06)	0.625		
Gender	0.30 (0.10, 0.84)	0.022	3.16 (1.10, 9.02)	0.032
Diabetes mellitus	2.26 (0.86, 5.93)	0.098		
Multivessel disease	5.19 (1.49, 18.05)	0.010	3.211 (1.21, 8.53)	0.019
LM-LAD QFR	2.20 (0.29, 16.34)	0.453		
LM-LCX QFR	4.44 (1.71, 11.51)	0.002	3.21 (1.21, 8.53)	0.019
Diameter stenosis (ostial LCx) (%)	1.04 (1.01, 1.08)	0.005		
Minimal lumen diameter (ostial LCx)	0.38 (0.16, 0.91)	0.031		

**Figure 2:**
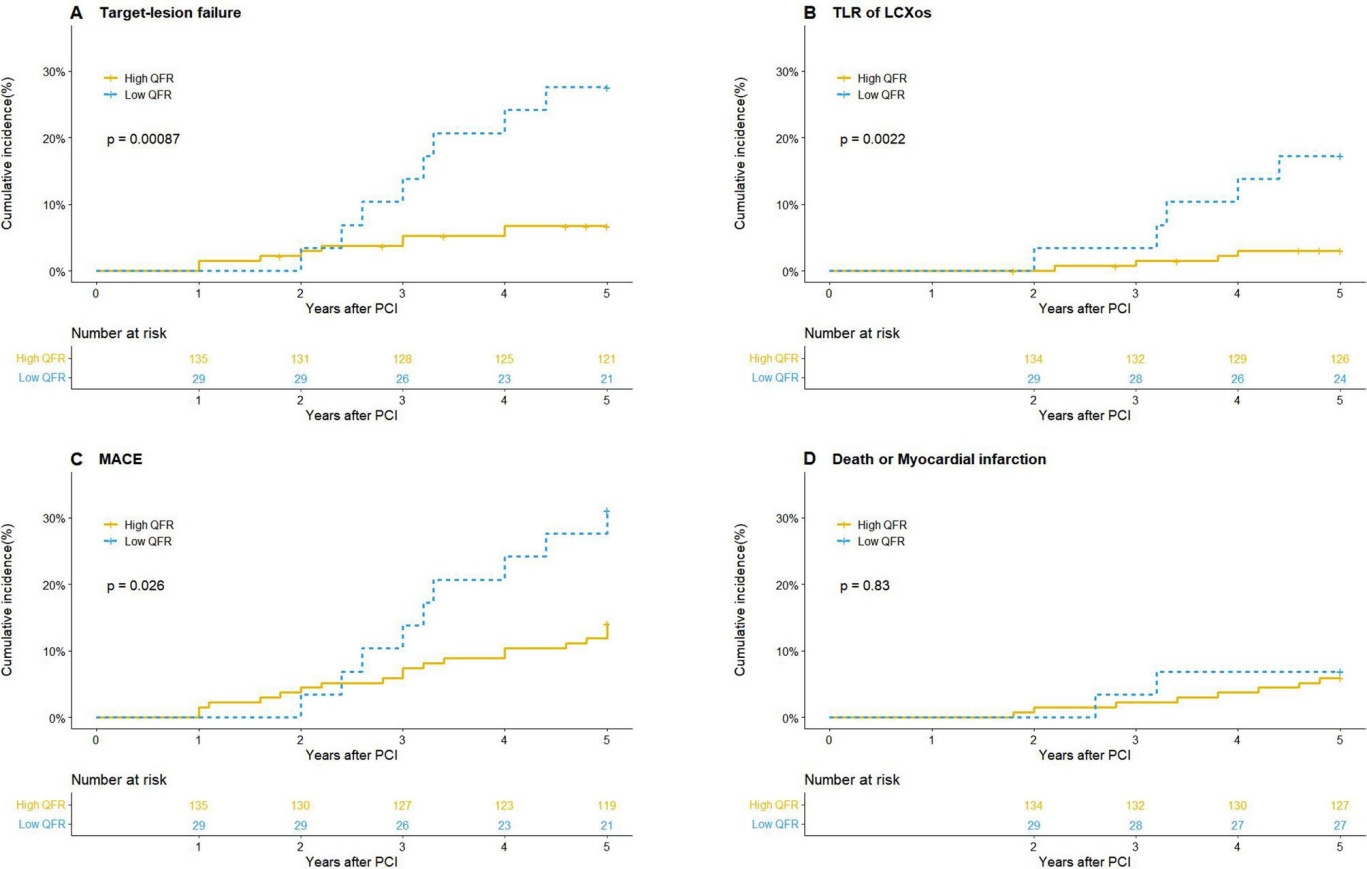
5-Year event rate according to QFR in jailed LCx after LM simple crossover stenting. Comparison of estimated event rates including: (**A**) target lesion failure, (**B**) target lesion revascularization of LCXos, (**C**) MACE, (**D**) death or myocardial infarction between the high QFR group (continuous line) and the low QFR group (dotted line).

**Figure 3:**
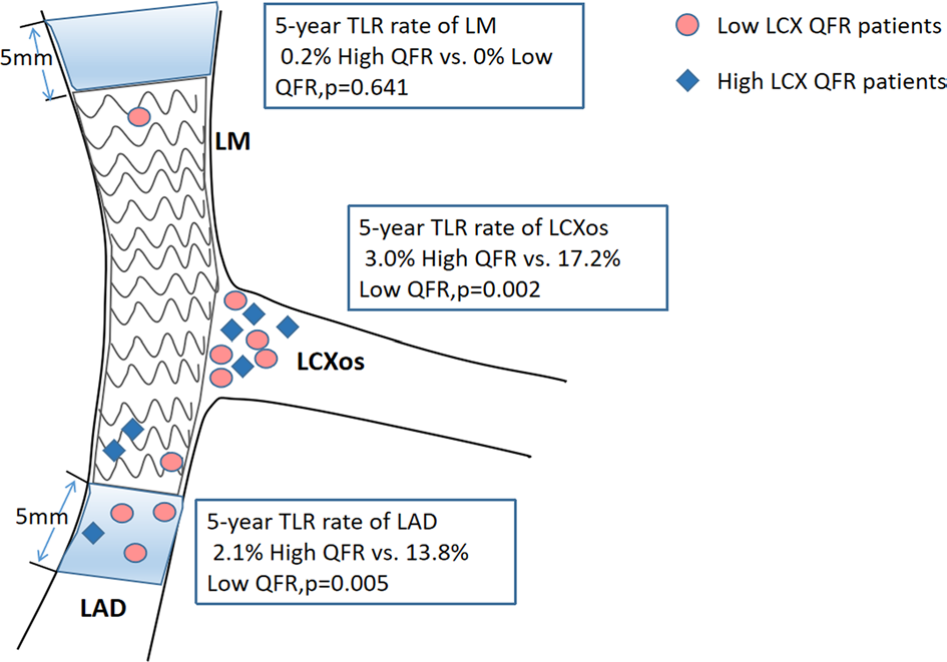
Comparison of target lesion revascularization (TLR) events occurred at different location. The Kaplan–Meier method was used to calculate the 5-year TLR rate at each location.

Compared with the high QFR group in the analysis of 5-year clinical outcomes, 5-year rate of TLF (27.6% vs. 6.7%; p = 0.0015) (Table 4, Fig. 2), 5-year LCx ostium-related TLR rate (17.2% vs. 3.0%; p = 0.002) (Figs. 2, 3) in the low QFR group was significantly higher. It had to be mentioned that 5-year TLR rate of proximal LAD was also higher in the low QFR patients (13.8% vs. 2.1%, p = 0.005). In addition, the event rates of TVF, repeat revascularization (including TVR and TLR) and MACE in low QFR group was also higher (all p < 0.05) (Fig. 2). Even after PSM, 5-year TLF, TLR of LCx ostium and MACE of low QFR group were still at higher risk (all p < 0.05), except for death or myocardial infarction (p > 0.05) (Fig. S1).

**Table 4.** 5-Year clinical outcomes according to QFR of jailed LCx.

	High QFR (n = 135)	Low QFR (n = 29)	P value	Hazard Ratio (95% CI)	P value
Target lesion failure	9 (6.7)	8 (27.6)	0.00087	4.24 (1.21, 14.95)	0.0015
TLR of LCXos	4 (0.03)	5 (0.172)	0.002	6.07 (1.63, 22.59)	0.007
Target vessel failure	11 (0.083)	8 (0.276)	0.003	3.68 (1.48, 9.15)	0.005
Death from any cause	7 (5.2)	0 (0)	0.215	–	–
Cardiac death	2 (0.015)	0 (0)	0.507	–	–
Noncardiac death	5 (0.037)	0 (0)	0.295	–	–
Myocardial infarction	2 (0.015)	2 (0.069)	0.09	4.66 (0.66, 33.05)	0.124
Repeat revascularization					
Target vessel	10 (0.075)	8 (0.276)	0.001	4.05 (1.60, 10.27)	0.003
Target lesion	8 (0.06)	8 (0.276)	< 0.001	5.06 (1.90, 13.48)	0.001
MACE	19 (0.141)	9 (0.31)	0.026	2.39 (1.08, 5.27)	0.032

**Figure 4:**
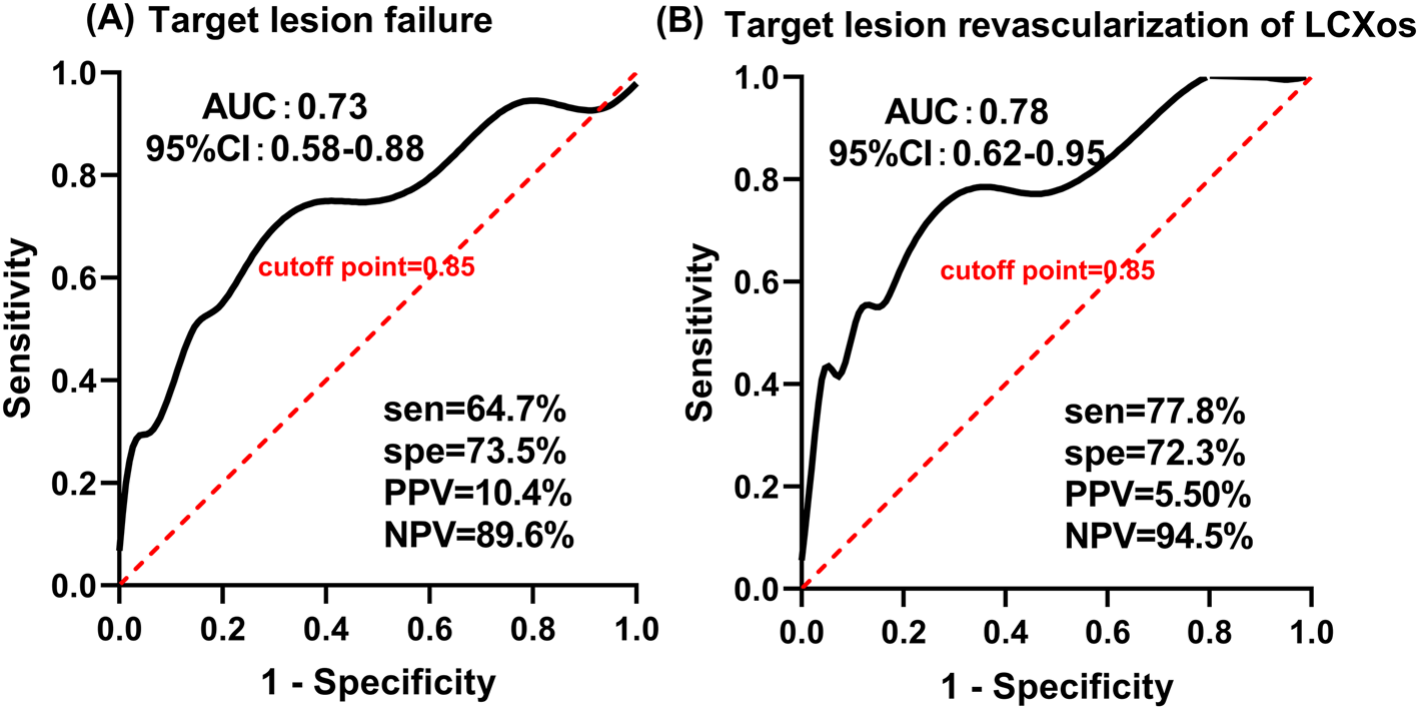
(**A**) The QFR in jailed LCx after LM simple crossover stenting predicting 5-year TLF; (**B**) the QFR in jailed LCx after LM simple crossover stenting predicting 5-year TLR of osial LCx.

ROC analysis was conducted to evaluated the the efficiency of QFR in prediction for 5-year clinical outcomes. The cut-off value, QFR < 0.85 of the jailed LCx, predicted 5-year TLF with a sensitivity of 64.7%, a specificity of 73.5%, a positive predictive value (PPV) of 10.4%, and a negative predictive value (NPV) of 89.6% (area under curve [AUC] 0.73, 95% CI 0.58–0.88, p < 0.05), and the percentage in prediction of 5-year TLR of LCx ostium was 77.8%, 72.3%, 5.5%, 94.6% (AUC 0.78, 95% CI 0.62–0.95, p < 0.05), respectively (as showed in Fig. 4).

## Discussion

In this retrospective cohort study addressing the impact of QFR measured in jailed LCx after successful LM simple crossover stenting on long-term clinical outcomes, we found that: (1) although a certain correlation existed between DS% and QFR in jailed LCx after LM crossover stenting, DS% could not predict 5-year clinical outcomes; (2) patients with low QFR (< 0.8) in jailed LCx after LM simple crossover stenting were at greater risk of 5-year TLF than those with a high QFR; (3) QFR measured immediately in jailed LCx may be helpful to identify the functional significance of the comprised ostium of LCx after LM-to-LAD stent implantation.

Similar to previous studies^9,21^, angiographic parameters like minimal lumen diameter and DS% of the jailed LCx, could not predict the clinical outcome. Angiography alone to guide treatment decision on the jailed SBs has proved to be unreliable in bifurcation lesions. Previous studies reported a poor correlation between FFR and angiographic DS% in jailed SBs after MB stenting^5,9,22^. Ahn observed that, in total 230 non-LM bifurcation lesions, only 28.4% of SBs with angiographic DS ≥ 50% had FFR ≤ 0.80, and 13.5% of with angiographic DS < 50% had FFR ≤ 0.80^23^. Similar results was found in the current study that among 24 SBs with ≥ 50% DS, 58.3% had QFR ≤ 0.80, which was relatively higher than previous studies, and only 10.7% of SBs with DS < 50% had QFR < 0.8. Slightly difference from previous study was that a relatively better correlation (R = − 0.67) exsited between QFR and angiographic DS% of the jailed LCx. Such difference may be explained by the following reasons: firstly, FFR often measured within 5 mm the ostial lesion of the jailed SBs, which reflects a local pressure drop. In contrast, computation of QFR for LM-LCX covered nearly the entire vessel from proximal to distal. In order to minimize the impact of non-ostial LCx lesions on the QFR measurement of jailed LCX ostium, significant non-ostial lesions were excluded in this study. In addition, the measurement of QFR itself is also based on angiographic views, so it has a more significant correlation with angiographic DS%. Of course, QFR is superior to angiography because it considers not only geometric boundary of coronary artery, but also hemodynamic informations such as blood flow velocity, pressure drop, microvascular resistance, etc^24^. Finally, the small sample size also might lead to biased results, so caution is required when interpreting our results.

Physiological evaluation indicators represented by FFR were validated by a series of investigations in the decision making for SBs interventions. It was reported that the FFR-guided SBs interventional strategy in non-LM bifurcation lesions did not improve clinical outcome^7,10^. Inconsistent result was found in LM bifurcation lesions, Lee's retrospective analysis indicated that the low FFR (< 0.8) in the jailed LCx after LM crossover stenting was at a higher risk of 5-year TLF rate^25^, which was explained by the reason that the jailed LCx had a relatively larger myocardial territory. QFR, as a novel approach for fast computation of FFR, has been applied in LM bifurcation lesions for the first time in present study. It was found that patients with a low QFR (< 0.8) in the jailed LCx after LM simple crossover stenting were at greater risk of 5-year TLF than those with a high QFR, mainly at the expense of more revascularization over the LCx ostium. This result suggeted the functionally jailed LCx defined by low QFR (< 0.8) had a significantly greater impact on the clinical outcome.

**Figure 5:**
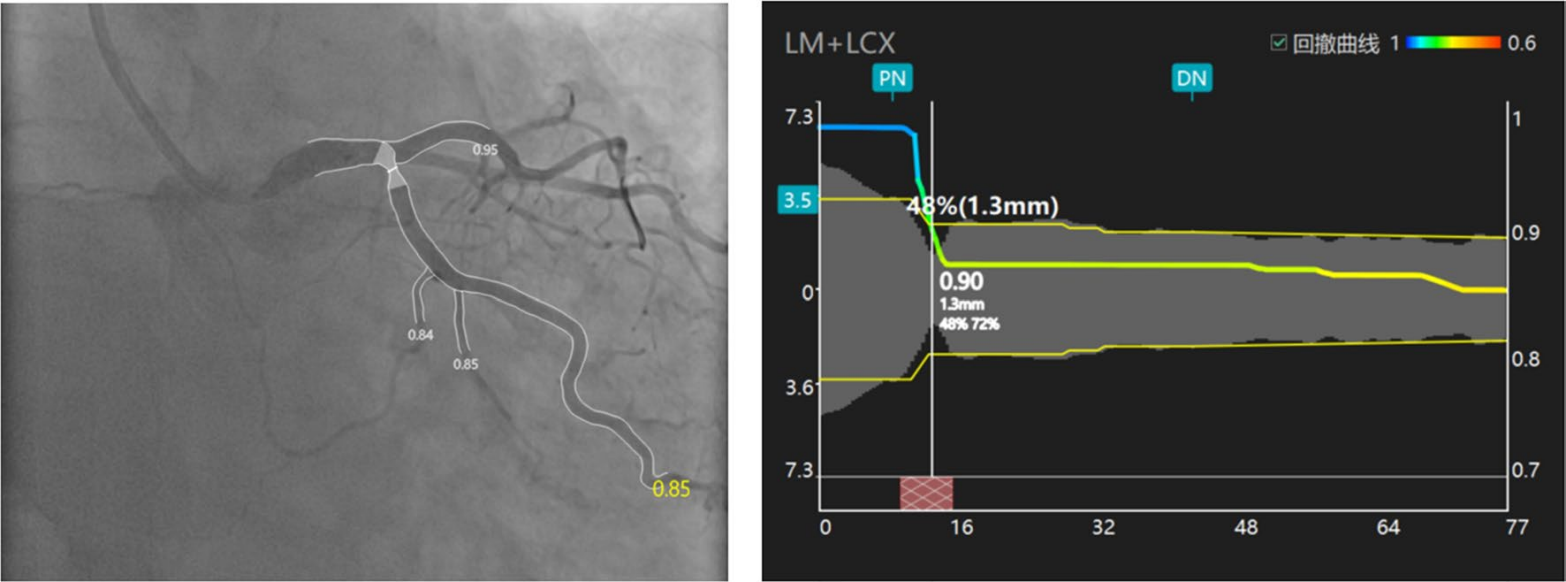
A representative example of computation of QFR. Left panel shows a stenosis at the ostium of LCX after LM-LAD stent implantation. The lumen contours of LM-LCX and its side branches were automatically delineated and superimposed on the angiographic images. The computed QFR was 0.85. Right panel shows the co-registration between lumen size and QFR pullback at every position along the LM-LCX.

In the bifurcation lesions, acquisition of two qualified angiographic views both with good exposure of SB ostium is actually difficult. The new method based on the Murray bifurcation fractal law, make it possible to compute QFR for the jailed LCx from a single angiographic view (as showed in Fig. 5). The jailed LCx with a QFR < 0.8 after LM crossover stenting was associated with long-term adverse events, the majority of which was the unsheduled TLR on the ostial LCx. In the multivariate analysis, the post-stent QFR in the jailed LCx was an independent predictor of the 5-year TLF. This result was further strengthened in ROC analyse, when QFR of the jailed LCx was < 0.85, there was only a small probability of predicting 5-year TLF or TLR of osial LCx (PPV = 10.4%, 5.5%, respectively), but post-stent QFR of the jailed LCx ≥ 0.85 could exclude the events of TLF or TLR in LCx ostium with a great possibility (NPV = 89.6%, 94.5%, respectively). These findings suggested us that QFR evaluation of the LCx after LM crossover stenting may be useful for operators to decide whether additional procedures are needed for the jailed LCx. If a QFR of the jailed LCx is low (< 0.8), a POT or final kissing balloon should be taken into consideration. POT symmetrically expanded the proximal and bifurcation segments of the stent, enlarging the strut cells, which ameliorating the FFR value of SB^26^. Side branch dialation, kissing balloon inflation also opened the strut cells but expanded the stent asymmetrically and might induce SB dissection requiring stenting of the SB^27^. Re-POT sequence(POT, SB dialation plus POT), rePOT sequence with/without kissing balloon effectively open the side branch struts and expands the stent, which improved clinical outcomes in the patients with a side branch FFR < 0.75^28–30^, whereas such sequence is a complex procedure. Conversely, if QFR is ≥ 0.85, additional procedures are not required, While it range from 0.8 to 0.85, as a grey zone, extra evaluation such as Intravascular ultrasound (IVUS) and optical coherence tomography (OCT) examination should be considered. Of note, the higher incidence of TLR at the proximal LAD in the low QFR group was found, the authors reviewed the angiographic views and found that all TLR events occurred at the margin of the stent, which may be caused by accelerated plaque progression in nontarget lesions after stent implantation. The underlying reason was explained by Ma J in his rabbit models that stent implantation triggered acute phase response and systemic inflammation, contributing to plaque growth and instability^31^. In addition, although no significant difference was observed, the length of LM to LAD lesions was indeed longer in baseline data, which further indicated that the low QFR patients suffered heavier plaque burden.

## Study limitations

First, our study selected patients exhibiting a good angiographic morphology without any significant LCx disease, which was not representative true LM bifurcation disease. Furthermore, the inclusion criterions required LCx ≥ 2.5 mm, which was a very high selectivity, brought about selection bias. Second, intracoronaery imaging information (e.g. IVUS and OCT) of bifurcation lesions have not been collected, which provided more data on the plaque progression and carina shift, and may figure out the exact cause of SB compromise. Third, the sample capacity is limited, the number of subjects with low QFR was modest (29), but the baseline characteristics did not exhibit a difference between the two groups, therefore, we considered the statistical power would not fade significantly. Fourth, the computation of QFR depends on an angiographic view. It was not always possible to obtain the optimal view because of vessel overlap, tortuosity and insufficient intra-coronary contrast-media injection, which may affect the accuracy of QFR measurement. Despite these limitation mentioned above, our data provided some previously unreported evidence for QFR-guided SB intervention strategy in bifurcation lesions and could play a hypothesis-generating role for future research.

## Conclusions

The present study demonstrates the long-term prognostic implications of the QFR-based functional assessment in the jailed LCx after LM crossover stenting. The low QFR (< 0.8) measured in jailed LCx was significantly associated with worse 5-year clinical outcomes after LM crossover stenting. Hence, the QFR may offer a novel tool to advance risk stratification and guide therapeutic decision on whether additional procedures is needed for the jailed LCx after LM crossover stenting.

## Supplementary Information

Supplementary Information.

## Data Availability

The datasets used and/or analyzed during the current study are available from the corresponding author on reasonable request.

## Abbreviations

QFR: Quantitative flow ratio
FFR: Fractional flow reserve
TLF: Target lesion failure
PCI: Percutaneous coronary intervention
QCA: Quantitative coronary analysis
TLR: Target lesion repeat revascularization
TVF: Target-vessel failure
TVR: Target vessel revascularization
MACE: Major adverse cardiac event-composite endpoint
POT: Proximal optimizing technique
