# Supplementary material for: Long-term prognostic value of Murray law-based quantitative flow ratio in jailed left circumflex coronary artery after left main crossover stenting
Source: Sci Rep. 2023 Mar 16;13:4391. doi: 10.1038/s41598-023-30991-4 (PMC10020166; doi:10.1038/s41598-023-30991-4)
Supplement: Supplementary file 1 — Supplementary Information. [file 41598_2023_30991_MOESM1_ESM.docx]

1. **Supplemental method**

**Detail of QFR computation and quantitative coronary angiography measurement**

The computation of QFR was summarize as the following steps, if more detailed steps was needed, please refer to Prof. Tu's article^[[1]](#endnote-1)^: (a) delineation of the interrogated LM-LCx or LM-LAD during contrast injection and calculation of contrast flow velocity based on the centerline length divided by the contrast dye-filing time; (b) selection of the analysis frame with sharp lumen contour at the stenotic segment as key frame; (c) delineation of the lumen contour of the interrogated vessel and its SBs with diameters of ≥1.0 mm on the key frame; (d) reconstruction of reference diameter function with step-down size across bifurcations according to Murray fractal law; (e) AngioPlus software automatically calculates QFR. A quantitative coronary analysis (QCA) data on 3 segments (the proximal main vessel, distal mainvessel, and SB) were also collected. The variables measured included the reference diameter, minimal lumen diameter, lesion length, and percentage of diameter stenosis .

1. **Supplemental tables**

Table S1 Baseline characteristics of patients in the propensity score matched (PSM) cohorts

|  | All patients(n=116) | High µQFR(n=87) | Low µQFR(n=29) | P value |
| --- | --- | --- | --- | --- |
| Age,yrs | 64.76±9.73 | 65.69±9.33 | 61.97±10.514 | 0.074 |
| Men | 12(10.3) | 8(9.2) | 4(13.8) | 0.491 |
| Hypertension | 76(65.5) | 59(67.8) | 17(58.6) | 0.376 |
| Diabetes mellitus | 49(42.2) | 35(40.2) | 14(48.3) | 0.517 |
| Insulin treatment | 11(9.5) | 9(10.3) | 2(6.9) | 0.728 |
| Hyperlipidemia | 31(26.7) | 21(24.1) | 10(34.5) | 0.334 |
| Current smoker | 70(60.3) | 51(58.6) | 19(65.5) | 0.662 |
| Previous MI | 43(37.1) | 30(34.5) | 13(44.8) | 0.377 |
| Overweight(BMI≥24） | 55(47.4) | 42(48.3) | 13(44.8) | 0.831 |
| Ejection fraction,% | 65.8(55.93,68.88) | 66.0(58.1,69.3) | 61.1(48.0,68.0) | 0.172 |
| NT-proBNP,pg/ml | 141.0(56.25,527.5) | 135(56.0,548.0) | 156.0(62,449.5) | 0.821 |
| Clinical presentation |  | | | |
| Stable angina | 19(16.4) | 15(17.2) | 4(13.8) | 0.456 |
| Acute cornary syndrome | 97(83.6) | 72(82.8) | 25(86.2) |  |
| STEMI | 14(12.0) | 10(11.5) | 4(13.8) |  |
| Non-STEMI | 45(38.7) | 31(35.6) | 14(48.2) |  |
| Multivessel disease | 64(55.2) | 45(51.7%) | 19(65.5%) | 0.281 |

TABLE S2 Lesion and procedural characteristics in the PSM cohorts

| Baseline | All patients(N=164) | High µQFR(n=87) | Low µQFR(n=29) | P value |
| --- | --- | --- | --- | --- |
| LM-LAD |  | | | |
| Reference vessel diameter, proximal, mm | 3.2(3.0,3.5) | 3.2(2.9,3.5) | 3.2(3.0,3.5) | 0.742 |
| Reference vessel diameter, distal, mm | 2.5(2.3,2.8) | 2.5(2.3,2.8) | 2.5(2.35,3.0) | 0.166 |
| Minimal lumen diameter, mm | 1.3(1.0,1.6) | 1.3(1.0,1.6) | 1.40(1.05,1.65) | 0.445 |
| Diameter stenosis, % | 52.0(43,61.0) | 53.0(45.0,62.0) | 48.0(38,59.5) | 0.118 |
| LM-LAD lesion length,mm | 18.2(12.5,28.1) | 17.2(12.1,27.4) | 21.0(14.3,34.1) | 0.314 |
| LCx |  | | | |
| Reference vessel diameter, distal, mm | 2.7(2.6,3.0） | 2.7(2.6,3.0) | 2.7(2.9,2.5) | 0.167 |
| Minimal lumen diameter, mm | 2.4(2.0,2.7) | 2.4(2.08,2.70) | 2.2(1.8,2.55) | 0.055 |
| Diameter stenosis, % | 16.0(5.0,27.0) | 16.0(5.0,25.7) | 21.0(5.0,42.0) | 0.316 |
| LM-LAD stent |  |  |  |  |
| stent length, mm | 24.0(18.0,30.0) | 24.0(18.0,30.0) | 24.0(18.0,31.5) | 0.962 |
| Stent diameter, mm | 3.5(3.5,4.0) | 3.5(3.5,4.0) | 3.5(3.0,4.0) | 0.928 |
| **After LM-LAD stenting** | | | | |
| LM-LAD |  | | | |
| Minimal lumen diameter, mm | 2.9(2.3,3.2) | 2.8(2.3,3.2) | 3.0(2.4,3.2) | 0.371 |
| Diameter stenosis, % | 10.5(1.30,20.75) | 12.0(3.2,22.0) | 4.0(0,16.5) | 0.093 |
| LM-LAD QFR | 0.96(0.93,0.98) | 0.96(0.93,0.98) | 0.94(0.92,0.98) | 0.393 |
| Ostial LCx |  |  |  |  |
| Minimal lumen diameter, mm | 1.8(1.5,2.2) | 1.9(1.5,2.3) | 1.6(1.10,1.80) | <0.001 |
| Diameter stenosis, % | 35.0(27.0,46.75) | 33.0(24.0,42.0) | 46.0(32.5,57.0) | <0.001 |
| LM-LCx QFR | 0.865(0.79,0.93) | 0.89(0.86,0.94) | 0.74(0.68,0.77) | <0.001 |

| Table S3 Univariate and multivariate cox proportional hazard analyses for 5-year TLR of osLCX in the PSM cohorts | | | | |
| --- | --- | --- | --- | --- |
|  | Univariate | | Multivariate | |
|  | HR (95% CI) | p Value | HR (95% CI) | p Value |
| Diabetes mellitus | 10.43(1.28,8.76) | 0.028 | 9.58(1.18,78.06) | 0.035 |
| Minimal lumen diameter(ostial LCx) | 1.17(0.03,0.91) | 0.038 |  |  |
| LM-LAD QFR | 0.002(0.001,3.003) | 0.086 |  |  |
| Multivessel disease | 6.00(0.74,48.80) | 0.094 |  |  |
| LM-LCX QFR | 5.21(1.25,21.82) | 0.024 | 4.68(1.12,19.63) | 0.035 |

| Table S4 Univariate and multivariate cox proportional hazard analyses for 5-Year TLF in the PSM cohorts | | | | |
| --- | --- | --- | --- | --- |
|  | Univariate | | Multivariate | |
|  | HR (95% CI) | p Value | HR (95% CI) | p Value |
| LM-LCX QFR | 4.37(1.51,12.59) | 0.006 | 0.002(0.001,0.18) | 0.007 |
| Diabetes mellitus | 2.57(0.86,7.67) | 0.091 |  |  |
| Multivessel disease | 5.28(1.18,23.58) | 0.029 |  |  |
| Minimal lumen diameter(ostial LCx) | 0.15(0.04,0.53) | 0.003 | 0.28(0.08,0.95) | 0.041 |

1. **Supplemental figures**

| FIGURE S1 5-Year event rate according to QFR in jailed LCx after LM simple crossover stenting in the PSM cohorts | |
| --- | --- |
| 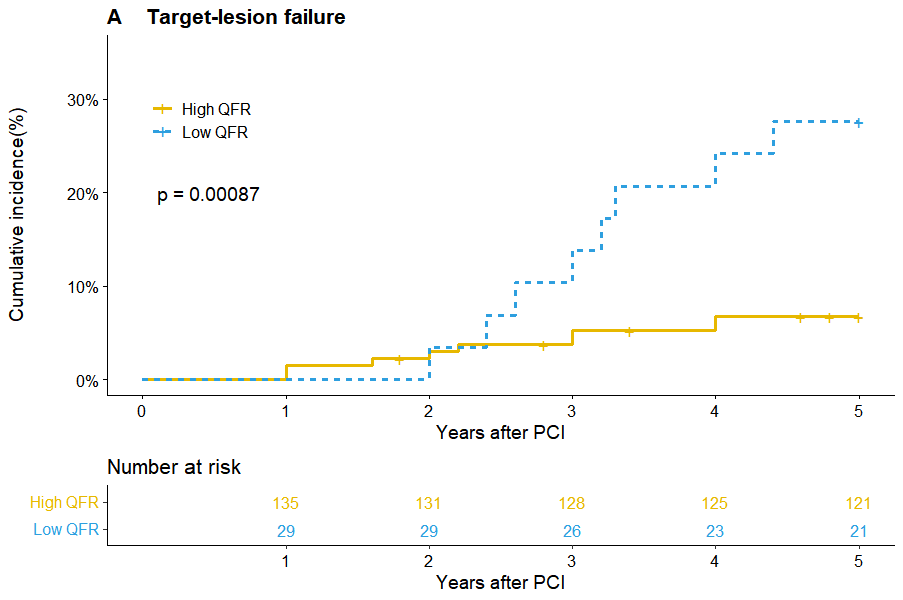 | 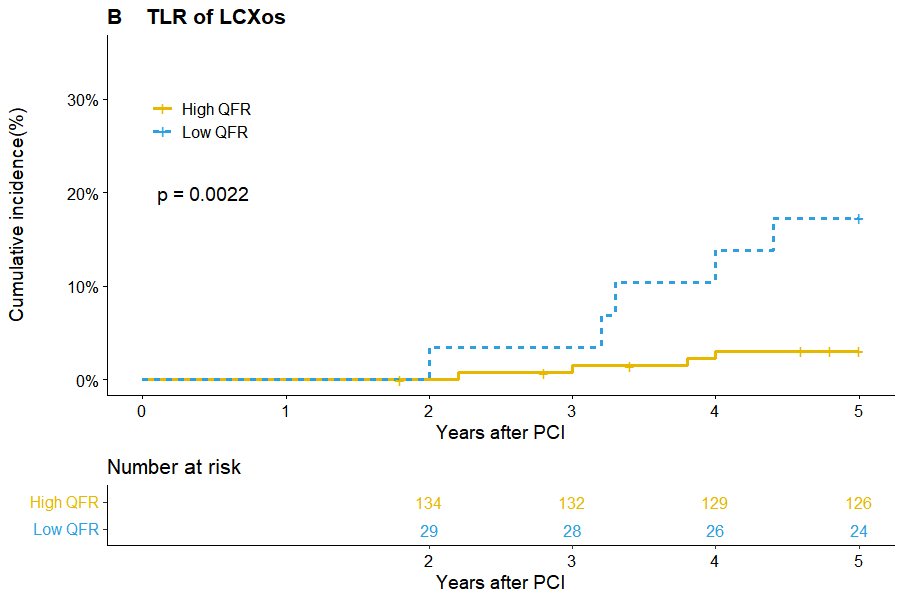 |
| 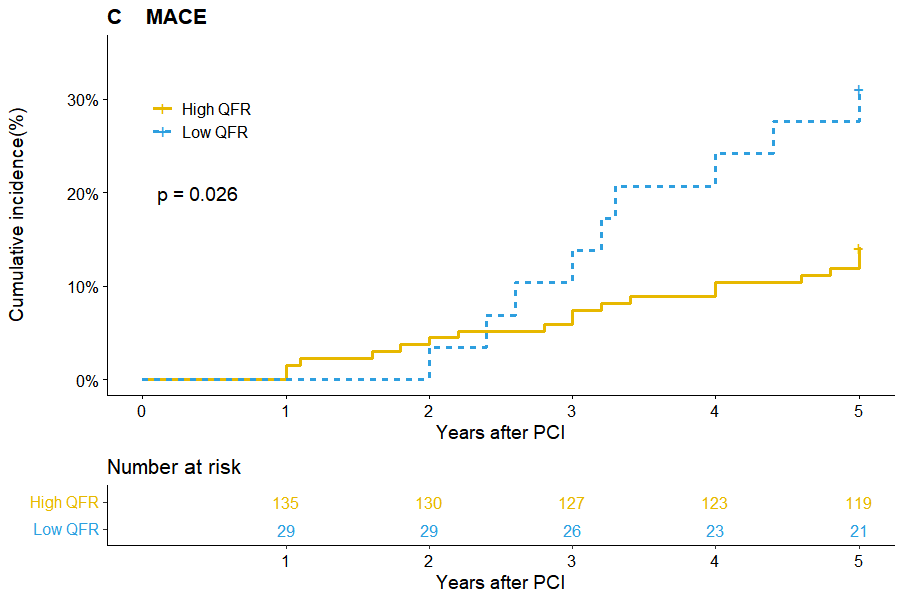 | 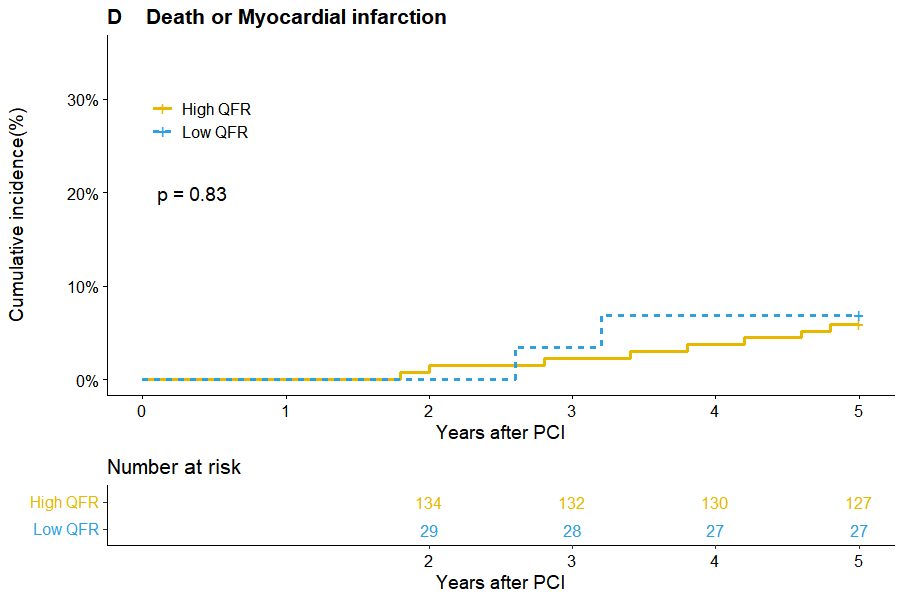 |
| Comparison of estimated event rates including: (A) Target lesion failure, (B) Target lesion revascularization of LCXos, (C)MACE, (D)Death or myocardial infarction between high µQFR group (continuous line) and low µQFR group (dotted line) in propensity score-matched cohorts. | |

1. Tu S, Ding D, Chang Y, Li C, Wijns W, Xu B. Diagnostic accuracy of quantitative flow ratio for assessment of coronary stenosis significance from a single angiographic view: A novel method based on bifurcation fractal law. Catheter Cardiovasc Interv. 2021 May 1;97 Suppl 2:1040-1047. [↑](#endnote-ref-1)
